# Sexual harassment and sexual assault in the Western Australian mining industry: a qualitative examination of the perceptions of key stakeholders

**DOI:** 10.3389/fpubh.2024.1432990

**Published:** 2024-08-08

**Authors:** Sarah Vrankovich, Sharyn Burns, Cheryl M. H. Yam, Sharon K. Parker, Jacqueline Hendriks

**Affiliations:** ^1^School of Population Health, Curtin University, Bentley, WA, Australia; ^2^Centre for Transformative Work Design, Future of Work Institute, Curtin University, Bentley, WA, Australia

**Keywords:** mining, harassment, assault, sexual violence, qualitative data, Australia, workplace

## Abstract

**Introduction:**

The prevalence, pervasiveness, and minimization of sexual harassment and sexual assault (SHSA) within the Western Australian mining industry has been revealed in recent Australian reports and inquiries. However, there remains a gap in scholarship focusing on SHSA within the mining sector, specifically that engages with mining employees to understand this issue.

**Methods:**

This study aimed to fill this gap by exploring the experiences and perspectives of Western Australian mining employees in relation to SHSA. Through qualitative research methods, stakeholders (*n =* 30) from various industry roles (e.g., front-line operations, administrative staff) participated in semi-structured interviews. A deductive thematic analysis was conducted to analyze the data.

**Results:**

The study revealed diverse perspectives of SHSA among participants, highlighting how this issue is understood, addressed, and discussed within the workplace. While some participants recognized positive shifts in workplace culture, it became apparent that additional efforts are needed to tackle the underlying and persistent factors that contribute to sexism, misogyny, and, ultimately, SHSA. Participants shared their perspectives regarding strategies and initiatives that could effectively combat SHSA within the industry.

**Discussion:**

This study constitutes a significant contribution to the limited body of research on SHSA in the Western Australian mining sector, offering valuable insights and recommendations for future prevention initiatives.

## 1 Introduction

The mining industry is one of many sectors that has garnered global attention for its widespread and persistent, yet often minimized, concerns of sexual harassment and sexual assault (SHSA) in workplaces (1–4). Studies have consistently shown that women working in the mining industry face heightened levels of SHSA, while knowledge about men’s experiences remains limited (1, 3–6). The industry’s working culture, characterized by its masculine ethos and predominantly male workforce, is known to contribute to the prevalence of SHSA (4).

Fitzgerald’s Tripartite Model of Sexual Harassment defines sexual harassment as an umbrella term encompassing three intertwined domains: unwanted sexual attention, coercion, and gender harassment (7, 8). Unwanted sexual attention includes comments about a person’s body, catcalling, leering, unwanted sexual touching, following, or repeated requests for dates. Coercion involves demanding sexual favors or contact in exchange for benefits or rewards, such as employment or favorable work conditions. Gender harassment refers to sexual gestures, sexist or heterosexist language, and behaviors or attitudes that insult, intimidate, or degrade based on gender, identity, or sexual orientation (7, 8). This framework helps understand and address various forms of workplace sexual harassment. Additionally, sexual assault involves any sexual contact or intent of contact without consent, using physical force, intimidation, or coercion (9).

The challenges that specifically women encounter in the mining industry in relation to SHSA has been explored (4). In a Canadian study, women working in the oil and gas industry spoke about changing their behaviors to become “tough” and meet the industries demand for less feminine traits (10). In another example, a mixed method study (*n* = 87) with women mining stakeholders who held various positions across multiple countries, predominantly Ghana and the USA, completed an online survey and participated in an interview (4). Survey participants reported to have experienced gender-based discrimination (53%) and sexual harassment (37%). Significantly, 51% of those who reported discrimination or harassment experienced various forms of organizational retaliation, such as reduced income, job loss, demotion, and department reassignment. Other interpersonal barriers women can face when reporting include skepticism about the effectiveness of reporting and doubts regarding support from senior staff (11, 12). These findings highlight the pervasiveness and underreported nature of sexual harassment within the industry and the need for comprehensive interventions to ensure the safety and wellbeing of all mining employees.

In recent years, the issue of SHSA within the Australian mining industry has gained prominence. In 2022, the Australian Human Rights Commission (AHRC) released *Time for respect: Fifth national survey on sexual harassment in Australian workplaces* (13). This survey was conducted using online and telephone methods and involved over 10,000 participants. It revealed that over the preceding 5 years, 33% of Australians had experienced workplace sexual harassment, and prevalence within the mining industry was 32%. Gender disparities were evident within the mining industry, with 62% of women estimated to have experienced sexual harassment within the industry over the previous 5 years, compared to 25% of men (13). This indicates that women are more likely to experience sexual harassment, highlighting the prevalence of gender-based harassment within the industry, as described in Fitzgerald’s Tripartite Model (7, 8).

Numerous inquiries and investigations have highlighted the pervasive nature of SHSA within the mining industry. A national example includes the AHRC’s Respect@Work: *National Inquiry into Sexual Harassment in Australian Workplaces* (14). Specific to the Western Australian context is Rio Tinto’s release of an external review on workplace culture in the *Everyday Respect, Report into Workplace Culture at Rio Tinto* (15), and the Western Australian Parliament’s Community Development and Justice Standing Committee’s inquiry, *Enough is Enough: Sexual Harassment against Women in the FIFO Mining Industry* (16). The *Enough is Enough* report gathered evidence from 87 written formal submissions, including those from government and industry agencies, professionals, politicians, and individuals with lived experience of SHSA within the mining industry. It highlighted the widespread presence of attitudes and behaviors among mining employees that perpetuate and normalize SHSA, including unwanted sexual attention, coercion, and gender harassment (7, 8). Central to the findings in this review, along with Rio Tinto’s *Everyday Respect* Report (15), is the imperative for transformation across company, industry, regulatory, and legislative levels (16).

Various risk factors across Fitzgerald’s Tripartite Model (7, 8) contribute to the prevalence of SHSA within the Western Australian mining industry (17). Despite ongoing initiatives for gender equality, the industry remains predominantly male-dominated with a particularly masculine workforce, where women are notably underrepresented in senior leadership positions (14, 16). International research highlights experiences of gender-based discrimination and sexual harassment towards women are particularly prominent in male-dominated workplaces (18, 19). Additionally, fly-in-fly-out (FIFO) work is commonplace in the Western Australian mining industry, with an estimated 60,000 employees being FIFO workers (20). This work introduces specific challenges to addressing SHSA within the Western Australian context. Factors such as work-provided accommodations, remote work conditions, liminal-timed work rosters, and the accessibility of alcohol in workplace settings have also been identified as impactful elements in understanding and addressing SHSA within this industry context (14, 16, 21).

Although there have been notable improvements around the awareness of sexual harassment, more sensitive issues like sexual violence remain ignored (22). While employers generally acknowledge the necessity of providing employees with training on topics like sexual harassment, training provided has typically been single, isolated sessions that are delivered during induction in an online module format, in which employees are informed about specific behaviors that are unacceptable on a worksite. Research highlights that Western Australian mining employees are often provided restricted opportunities to receive training that will be most effective to addressing the risks associated with SHSA (22).

There remains minimal recent scholarship on understanding SHSA within the Western Australian mining industry (16, 22). Of the recent literature focusing on sexual harassment within this context, much has focused on specific communities (23, 24). There is a dearth of research that engages with mining employees at all levels, such as FIFO workers and professional staff working residentially in office roles, to gather their experiences and promote their role as key stakeholders in addressing this issue. This research sought to fill this gap by exploring the complexities surrounding SHSA in the Western Australian mining context, examining various dimensions of this issue and proposing strategies for meaningful intervention and change and posed the research question: *what are the experiences of Western Australian mining industry workers in relation to sexual harassment and sexual assault within their workplace*?

## 2 Methods

### 2.1 Data collection

Participants were recruited through a wider project that sought to increase understandings of workplace culture and wellbeing within the Western Australian mining industry (25). A baseline survey on workers’ experiences in the industry prompted participants to provide their contact details if they wished to take part in an interview. Interested participants were randomly assigned to one of three interview topics, including SHSA. Semi-structured interviews were conducted to allow for a conversational discussion, while also ensuring rich data was collected. The interview guide (see Appendix A) was informed by the literature and interviews focused on gathering the opinions, reflections, and experiences of mining employees about SHSA in their workplace. Suggestions for future improvements the mining industry could make to prevent and manage SHSA were also gathered. Interviews were conducted online via Microsoft Teams by two research assistants with experience in collecting qualitative data related to sexual violence, and both staff maintained reflective journals. Interviews were conducted at a time convenient to the participants, most were during work hours, although some participants opted to complete the interview during their rostered time off. Voice recordings were captured by Microsoft Teams and transcribed using the Otter.ai application. Interviews took between 25 min to 2 hours.

### 2.2 Data analysis

Simple deductive thematic analysis was performed on the transcript data using NVivo 12 software program. Thematic analysis is a widely used and flexible approach that allowed the researchers to identify and understand the perspectives and experiences participants (26–28). A deductive approach is a method of analysis using pre-determined categories. Based on the research questions of this project, the following themes were identified: (1) sexual harassment and sexual assault in the mining industry; (2) workplace culture; (3) workplace structure and environment; and (4) strategies and programs focused on prevention and response.

In line with Braun and Clarke’s (26, 27) approach, the process of thematic analysis involved various stages. To determine credibility and maintain dependability (28), the research team familiarized themselves with the data by reading each transcript several times. One member of the research team initially identified meaningful units of data, or topic codes, in the transcripts which supported the pre-determined themes. To enhance confirmability (28), the topic codes were synthesized and categorized into over-arching themes. The identified themes were discussed and confirmed with the broader research team. Reflexive processes were also undertaken. The research team acknowledges that their perspectives as women working in the public health sector could influence their approach to data collection, analysis, and interpretation. While they have experience in sexual health, workplace, and mining-related research, none have worked in the mining industry. Reflexivity was conducted by reading and re-reading the transcripts, going between the data, codes and themes across the transcripts, and discussing each stage of this process with the research team (29). Direct quotes from the interviews are provided to help illustrate each of the themes. Some quotes were lightly edited for readability purposes; however, the meaning of each quote remains unchanged.

### 2.3 Ethical considerations

The Curtin University Human Research Ethics Committee approved this research (HREC2022-0502). Participants were provided with a Participant Information Sheet and given the opportunity to ask questions about the research via email and before the interview commenced. Written informed consent was provided by each participant prior to their participation in an interview.

Personal information and details about current workplaces were collected from all participants to ensure the research team gathered data from a diverse sample according to both their identity and workplace experiences. All data was de-identified to provide anonymity for workplaces and participants, and pseudonyms were used. A distress protocol was developed and followed throughout the interview process and all participants were provided support contacts.

## 3 Results

All interview participants (*n* = 30) were current employees of the mining industry in Western Australia, and job roles varied from corporate office positions to operations on mining sites. A few were not directly employed by a specific mining company as principal employees but worked in roles that connected them directly to a mining site, such as a contractor or labor hire (e.g., boiler maker). Most participants had worked in the mining sector for numerous years (R = 1–45 years, x = 13 years). At times, participants had been employed in a range of roles across small to large organizations throughout their mining careers. Table 1 provides a summary of participant demographic information. All overarching themes and sub-themes have been summarized in Table 2.

**Table 1:** Summary of mining employee’s demographic information.

Pseudonym	Gender identity	Fly in-Fly Out	Age (in ranges)	Years of service (in ranges)	Job role	Mining organization size^a^
Charlie	Man	Yes	41–50	11–15	Senior leader; operations	Medium
Tony	Man	Yes	51–60	6–10	Maintenance; planning	Large
Bob	Man	No	51–60	31+	Engineer	Large
Joe	Man	Yes	31–40	1–5	Medical; site-based intervention	Medium
Lilly	Woman	Yes	31–40	1–5	Specialist; communications	Large
Beth	Woman	Yes	51–60	1–5	Operations; heavy machinery	Large
Liam	Man	Yes	51–60	21–25	Operations; heavy machinery	Large
Jessie	Woman	Yes	61–70	31+	Leader; administration, flight schedule, catering, accommodation	Medium
Ruby	Woman	No	25–30	1–5	Strategic integration	Large
Jordana	Woman	Yes	51–60	26–30	Advisor; metal extraction	Large
Mia	Woman	Yes	31–40	16–20	Field supervisor	Small
Emma	Woman	Yes	25–30	6–10	Coordinator; junior training	Large
Olivia	Woman	No	41–50	1–5	Purchasing	Large
Blair	Gender neutral	Yes	31–40	11–15	Operations; mechanic	Large
Isla	Woman	Yes	41–50	16–20	Supervisor; controls	Medium
Freya	Woman	Yes	41–50	11–15	Senior leader; health and safety	Large
Hugo	Man	No	61–70	1–5 (20+ for oil and gas)	Senior leader; strategic planning	Large
Leo	Man	Yes	31–40	11–15	Operations; power station	Large
Valerie	Woman	Yes	51–60	16–20	Operations; heavy machinery	Large
Levi	Man	Yes	61–70	26–30	Executive advisor; health and safety	Medium
Phoebe	Woman	Yes	41–50	6–10	Operations; heavy machinery	Large
Morgan	Man	Occasional FIFO	41–50	11–15	Risk and safety	Large
James	Man	No	51–60	11–15	Operations; airport controls	Large
Alex	Man	Yes	31–40	6–10	Technician	Large
Clara	Woman	Yes	31–40	6–10	Senior leader; geology	Small
Alice	Woman	No	31–40	6–10	Senior leader; environment consultant	Large
Amelia	Woman	No	41–50	16–20	Senior leader; site closure	Large
Eleanor	Woman	No	31–40	11–15	Senior leader; rail operations	Large
Lucy	Woman	No	41–50	6–10	Consultant; technology	Large
Steve	Man	No	41–50	6–10	Contractor; boiler maker	Various sizes

a There are no official guidelines on categorising mining organization size. The classification of organization size has been informed by participant perspectives and information provided by the Department of Mines, Industry Regulation and Safety.

**Table 2:** Summary of themes and sub-themes.

**Sexual harassment and sexual assault in the mining industry**
Disparate perspectives regarding the prevalence of SHSA
Ability to define and identify SHSA varies
**Workplace culture**
Shift towards a positive culture: An industry striving to improve
Misogyny and sexism occur, and is often covert
Reacting to media spotlight versus a proactive response
**Workplace structure and environment**
Gender balance
Alcohol consumption
The “type” of work
The “types” of people who work in the mining industry
Infrastructure and environmental challenges
**Strategies and programs focused on prevention and response**
The effectiveness of in-person training
Understanding policy
Management of SHSA
Reporting experiences and pathways.
Unfair dismissal of sexual harassment.
Reporting of incidents to the broader workplace.

### 3.1 SHSA in the mining industry

#### 3.1.1 Disparate perspectives regarding the prevalence of SHSA

There was a dichotomy in how participants characterized SHSA, explaining it as either “common” or “uncommon” within their workplaces. Notably, it was primarily men who described SHSA as less prevalent in their work settings. Regardless, people who believed SHSA was uncommon justified this idea based on their personal experiences or observations. For instance:

“I definitely think there are cases of [SHSA] but to me, I haven’t heard a huge array of instances where it’s happening. I definitely don’t think it’s happening any more than any other workplace” (Lilly).

Similarly, although Liam strongly asserted that SHSA was “very uncommon” and that media exaggerated its pervasiveness, he also brought attention to another point:

“We don’t even know how many incidents there has been. I’ve only heard of two being reported in the area in WA over the last few years, you know, is it such a big thing?”

Similar sentiments were expressed by others:

“I haven’t personally seen anything, or I haven’t been involved in anything … it’s a broad brush of look at this, there’s so much sexual assault or sexual problems happening. It’s hard to gauge how accurate that is. I find it very hard to believe that there’s blokes walking around raping people on my sites … there might be guys who ogle over a pretty girl and maybe they think of that as oh they stared at me but that’s human nature” (Alex).

In contrast, some participants acknowledged the widespread occurrence of SHSA within the industry. Most women and a few men perceived sexual harassment as a common phenomenon and sexual assault as infrequent. There was a shared understanding that women encountered instances of sexual harassment frequently, although it was not always reported. For example:

“I can guarantee you that for every one of me, there’s thirty more that are not going to say anything. There’s not a female on a mine site that hasn’t been sexually harassed” (Isla).

A few participants shared their experiences of SHSA within the mining context as a victim/survivor or bystander. Their experiences covered a broad spectrum, including incidents that were formally reported and those that went unreported, ranging from subtle actions to more overt behaviors. For instance, Freya highlighted an incident she experienced of unwanted sexual attention and gender harassment during a half-an-hour presentation to a group of male colleagues:

“I was standing side on and what I didn’t realize is I had two buttons undone on my shirt and you could see absolutely everything … as soon as I walked out, one of my team mates told me … I walked straight back into the room and went so is there a reason nobody spoke up in here to tell me that both my buttons were undone and you could see my full cleavage and one of the guys said we didn’t want to say anything cause you know, it’s the best presentation we’ve ever had” (Freya).

Lucy also discussed her experience of unwanted sexual attention, recounting an incident involving a colleague whose behavior had progressively intensified over time. Initially, he asked her on a date, but his actions evolved into regular loitering around her desk at the end of the workday. Eventually, his behavior escalated to the point where he physically touched her:

“It was a tickle on my ribcage to the left of my breast coming out of a team meeting, done quickly and the guys next to me knew that something had happened … I couldn’t prove [the assault], but it happened, and it deeply affected me. I felt violated. I felt disrespected.”

Less commonly, men’s experiences of SHSA were shared. For example:

“There was a situation where a young man was being harassed by an older woman … she even tried to get into his tent, and he didn’t feel comfortable speaking to anyone about it, so he just left it” (Mia).

#### 3.1.2 Ability to define and identify SHSA varies

Broadly, all participants shared an understanding that sexual harassment encompassed unwelcomed attention, while sexual assault typically involved physical actions. Participants in managerial roles generally exhibited a greater grasp of the nuances of SHSA compared to frontline workers. For instance:

“Sexual harassment, what I think it means is just whistles and stuff like that, inappropriate things that you say to another person, male or female. And the assault is rape” (Liam, frontline worker).

“I think sexual assault is where crime has occurred in terms of the law, and that can usually be physical. I guess it’s mainly perpetrated by males onto females. It can take on a number of connotations. Sexual harassment is unwanted behavior of a sexual nature. And again, that can take on a lot more of a broader sort of range. It might be verbal, showing pictures, staring, or obviously joking and things like that, received by the person in a negative way and it’s of a sexual nature” (Charlie, senior leader).

Participants’ perception of SHSA prevalence appeared to be associated with their ability to appropriately define and identify it. This was exemplified by Leo, who worked on a mine site and initially said:

“I don’t think it’s common. Not at all, not on my site from what I’ve seen. The only thing that I do see here is brief comments, when it’s a group of guys.”

However, as the discussion unfolded, Leo detailed an incident during a training session where he encountered a colleague making sexualized remarks. He reflected:

“I don’t participate in the objectification of women, but it does still happen a lot. I did a training last month where one guy kept making all these different comments and I’m just thinking, he’s married, how’s your relationship with your wife?”

### 3.2 Workplace culture

#### 3.2.1 Shift towards a positive culture: an industry striving to improve

Some participants reported that their workplace fostered a positive culture. Those working in smaller organizations or internal teams frequently expressed this sentiment, and these factors seemed to facilitate close working relationships with colleagues.

Those who resided locally or held drive-in-drive-out positions expressed that an affiliation with a small town or regional center gave them a sense of belonging to the local community. This enhanced workplace culture by fostering a sense of belonginess and increasing awareness of potential consequences, that may spillover into their local communities, associated with harmful behaviors. For example, Olivia elaborated on how this local connection fostered a heightened sense of accountability:

“I think it’s a bit more accountability as well. If you’re going to run into someone that you will see at kids sports or at the Christmas party, I think it really creates a different culture.”

Overall, participants felt the mining industry was working towards improving conditions for employees. Most participants had seen a genuine move away from adverse conditions that would have negatively impacted on employees working in the industry. Specifically, participants of all genders contended that changes towards gender equity had positive and overt benefits for women. For example, Bob who had worked in the mining industry for 33 years reflected:

“When I first started my career, there was no such thing as an underground manager who was a woman. That was just unheard of. Now, I went to site the other day and there was two of them.”

Some women who had worked in the industry for many years drew comparisons between their initial experiences and the experiences that women entering the workforce today would face. Changes ranged from differing responses to discriminatory comments, to structural and clothing changes. For instance:

“I joined a new crew and told them I was a lesbian and one of the guys said, you’re only a lesbian because you haven’t had good sex yet. And if you didn’t laugh you at that, they’d think you’re a bit of a bitch, you’d get snuffed out. But I am not seeing that anymore” (Mia).

Here, Mia’s experience highlights improvements of gender harassment and unwanted sexual attention. Furthermore, there were recognized improvements to organizations having more diverse workforces and celebrating such diversity. For example:

“The workplace is very fair and equitable. We celebrate wins. It’s probably the only mining company that promotes diversity, we celebrate pride week, NAIDOC week, they don’t put up with any harassment” (Jessie).

Improved focus on employee wellbeing was noted. For example, Tony shared how in his experience, the pre-start meeting schedules had altered to include wellbeing checks with employees before the workday begins:

“There is a chat about how everyone’s feeling and whether we got a good sleep … They’re making sure that everybody’s feeling up to the job. People have opened up and said, I’m feeling a bit tired, and then they’ll be like, okay we’ll partner you up with someone for the day. At any point you can go to the supervisor and say if you’re feeling fatigued” (Tony).

The shift towards improving workplace conditions was seen to be due to improved knowledge about forms of discrimination in the industry and leadership teams becoming better equipped to disseminate knowledge about policies and procedures around SHSA to their teams. For example:

“It’s constantly moving and evolving to be a better place to work. I think the leaders are also receiving a lot more coaching from HR [human resources] and they’re more capable of addressing problems in the business” (Lucy).

#### 3.2.2 Misogyny and sexism occurs, and is often covert

Some participants asserted that the mining industry continues to struggle with entrenched hyper-masculinity and lingering sexist undertones. As described by Joe, alongside others, current workplace culture continues to shape and reinforce expectations about how men should conduct themselves within the industry:

“There is a norm of the way that men are expected to behave, which is probably towards a more misogynistic, over sexualization of everything … the language that’s used, or general discussions I’ve had in the workplace … or sexualizing colleagues who are of the gender that person is attracted to, things I don’t necessarily deem professional but as a man is deemed acceptable in the mining world” (Joe).

Recent efforts may have reduced obvious discrimination or harassment, but many participants argued that gender harassment had become more subtle or discreet. For example:

“I’m one of the more switched-on operation workers and I’m recognized as such by my leaders … it doesn’t help that every time there’s somebody new in the crew, they always will talk to me like I don’t know what the hell I’m talking about at first. They’ll always assume that I’m not a mechanic. Then when they know that I’m a mechanic, they always talk to me like I know nothing” (Blair).

Some participants who identified as women or non-binary discussed it was common for women to encounter resistance when they attempted to share their ideas or give directions. While contributions from a woman may have been overlooked, minimized, or dismissed, the same contribution from a man was actioned:

“As women, we have discussed at length the fact that we rely on procedures and written documents … that we can refer to at any point because we will be constantly challenged. We almost need to be able to quote it straight away so that the perception is that it’s not our opinion … we will be challenged if we don’t quote a policy or procedure, whereas the men in our team can just say XYZ and there’s rarely any push back” (Emma).

Expanding on this, Jordana described:

“I always feel that I have to deliver the better results, that if I say something wrong, or if I don’t deliver something to the quality that it has to be delivered at, a lot of times, I don’t have a second chance. They’ll just be like all good, this guy can solve it” (Jordana).

Shifting focus, unwanted sexual attention and personal appearance was noted by some participants as an important factor that impacted how an employee might be treated. For all genders, this sometimes resulted in gender-based harassment. In most instances this was an issue relevant for younger and/or “attractive” women. For instance, Phoebe explained:

“The challenge is simply being female. If you are young and attractive, you have your own set of challenges. If you are older and attractive, you have your challenges as well. If you’re unattractive, you’re left alone.”

A few participants argued that women should be cautious about wearing tight clothing or gym/leisurewear around site, or being overly focused on their appearance:

“Young people starting out, they don’t adjust to their environment. So, for example, wearing tight gym clothes to the mess where you’ve got a lot of experienced mining people or just blokes that have been away from home for weeks on end. You’ve just got to adapt and cover up and don’t wear [activewear brands] to the mess” (Alice).

However, for Levi, the need to be cautious about clothing applied to all genders:

“There was a photo of a young girl, fully made up in tight jeans with all the boots on and a yellow shirt. There should be female mentors who will take the person aside and say, do you understand that dressing like that, you’re baiting guys. And there are guys who do the same thing, you know, they’ll undo three buttons, show a bit of hair, make themselves smell pretty and do that and wander around and are baiting the women. So, there should be some mentors who actually identify these people and take them aside and say you’re baiting the other party … and then they get upset when someone does give you a tap on the butt” (Levi).

#### 3.2.3 Reacting to media spotlight versus a proactive response

The recent media coverage on SHSA in Western Australia was highlighted. Some thought this raised awareness and prompted mining organizations to address the issue. However, others expressed frustration that prevention strategies were hastily implemented in a “knee-jerk reaction” (Steve), without adequate planning. The extensive media coverage and response strategies resulted in a sense of isolation and labelling among some men:

“Everyone is tarred with the same brush. Just because of these incidents that have or have not happened at other sites, all people, including myself, are now rape and sexual deviants or whatever … the media, how would they like it if all media workers are thought of as rapists now” (Liam).

Participants proposed the recent media attention, along with the response by workplaces, impacted the ways employees interreacted with each other. Some participants of all genders noted a perceptible decrease in interactions between men and women that was driven by men’s cautiousness to avoid potential pitfalls:

“What I’m starting to see now is that there’s just an avoidance of talking to women in the workplace … why would you have a personal friendly chat with someone if you’re worried that you might say the wrong thing” (Alex).

This emerging pattern of “segregation” within mining sites was seen to be increasingly evident:

“You get the comments about male leaders not wanting to be alone or go for coffees with the females, but they’ll go for coffee with their men because they’re worried about blurring lines and what’s seen as appropriate … then they exclude the females or not connect with and help them with development in the same way they’re helping men because of their agenda” (Ruby).

“Because of the way our company has taken such a strong stand on any kind of mistreatment of females, or any kind of harassment and assault, I think a lot of the men up here are a little bit nervous to actually interact with women. They really are quite careful about what they say to me and how they say it until I build a rapport with them. I think that’s the biggest thing I’ve noticed is when a man comes to site that’s new, he seems to be a lot more easily integrated than a female would” (Lilly).

### 3.3 Workplace structure and environment

#### 3.3.1 Gender balance

It was evident mining organizations were making efforts to enhance gender diversity, however, participants reported that men still dominated the workforce. Participants expressed that if women were represented at all levels this would ensure a more balanced workforce which would enhance the likelihood of improved gender equality and institutional sexism. Phoebe spoke about how, if she was a man in the industry, her “life would be much easier” implying that women can face additional challenges related to gender-specific harassment:

“The same issues would still be there, but the sexual element would be removed … Women do not at all have proper representation in all areas of mining, and certainly not in where they are with their career and the older they get, the less opportunity that they have. The preference is towards the younger women.”

Participants discussed that the lack of women in leadership roles leads to power imbalance, impacting workplace culture. A few participants acknowledged that institutional sexism exists within the industry, as women often found themselves confined to junior roles while leadership positions were largely held by men, as Ruby mentioned:

“If we still have all the females being at the most junior levels, we’re never going to actually shift that power imbalance. We need to try to get more women into the leadership roles.”

Participants also noted how a skewed gender ratio can be particularly problematic in regional or remote worksites, leading to feelings of isolation and vulnerability:

“I don’t think that power dynamics are the same as in the city because there’s usually a 50/50 [gender] split. Whereas on site, it’s very male dominated. I think it’s very easy for any female to feel a little bit apprehensive” (Lilly).

“I suppose being a woman and being aware that I’m in a remote place with a bunch of guys who could physically overpower me at any point if they wished, you always have your guard up regardless of whether you’re an experienced person or not” (Clara).

The sentiments of the participants suggest that improving gender balance and ensuring representation, addressing institutional sexism, and considering the unique challenges of remote worksites are critical factors in preventing SHSA.

#### 3.3.2 Alcohol consumption

Changes in alcohol consumption patterns were observed following the implementation of various measures by mining organizations. For example, a few participants spoke about the introduction of a “four drink limit” and the availability of lower-percentage beers on site. This was mostly relevant to FIFO workers who resided in work-provided accommodation. Some participants viewed these measures as a temporary solution that may not effectively bring about a cultural transformation. Steve provided an analogy:

“To me [putting in alcohol restrictions] is like saying, we know that we have dangerous drivers, so we’re just going to have three-cylinder cars that can do a maximum of 60km/h.”

Some participants emphasized that alcohol consumption is a persistent issue within the mining industry that impacts the prevalence of unwanted sexual attention and gender harassment. As Levi expressed:

“One of the enabling factors in the industry is alcohol abuse. That certainly fuels probably 50 to 60% of these things.”

Amelia spoke about the influence of alcohol consumption during shift change. Firstly, she provided an explanation of what shift change is:

“Say, a two-week shift. Two weeks on, one week off. Midway through the swing, seven days in, they’ll go from doing day shifts, then they’ll have 18 hours off to go into night shifts.”

Amelia noted that during these shift changes employee’s “drink shit loads” to avoid detection of alcohol within their system at the start of their next shift. Similarly, Bob emphasized that alcohol consumption has been a variable to the prevalence of sexual harassment on sites. However, he argued this had been “considerably reduced”:

“The role alcohol plays on remote sites as a risk factor for all sorts of bad behavior, but sexual harassment in particular. This bad behavior is prevalent at shift change … where there is a 24-hour break from work. There has been a significant reduction in the availability of alcohol on FIFO sites over recent times and this has led to much better management of this abhorrent behavior”.

Others proposed that when alcohol is involved inhibitions are often lowered, which can contribute to a range of behaviors:

“When younger women go to the pub, there’s alcohol involved, which means inhibitions are less, women conduct themselves differently to men, we laugh, we talk … and sometimes I think that’s misconstrued by young men and they misread it … the young women seem to get kind of upset about that, but in actual fact, it was a genuine clumsiness on part of some men. And then there’s other men that just don’t know when to bugger off and let it go. And the more alcohol those type of people have, the worse it gets” (Beth).

#### 3.3.3 The “type” of work

The mining industry was described as a unique environment in which people’s livelihoods were closely intertwined with their employment. This was particularly evident in the context of FIFO work arrangements where there seemed to be “blurred lines between work and not work” (Joe). Participants noted that SHSA was highest among frontline operations workers, compared to those working in office settings, and among contract workers:

“Our underground contractors were pretty wild, and they would make sexual innuendos. They would print and try and send around sexual or naked pictures or things like that. It was a bit of a boy’s club, and it was kind of accepted and they’d just get told not to do it again” (Jessie, office management).

“I look after a big contracting group, it’s not just inappropriate behaviors, it’s probably safety in general. There’s normally a lower standard within those groups … their management system seems to be a little bit looser, and their HR departments are not as well resourced” (Charlie, frontline worker).

Here, participants noted that contract workers may have less perceived affiliation with an organization and may be less invested in fostering safe and respectful workplaces.

#### 3.3.4 The “types” of people who work in the mining industry

When exploring the factors influencing SHSA prevalence, disparate views about the traits of individuals attracted to working in the industry were discussed. For example:

“There’s a certain type of person that works in operations, it’s quite competitive and it’s very male dominated. It’s all about production, production, production … The company likes those sorts of people that have an aggressive nature … females aren’t considered in that group, they don’t have the same aggressive behaviors” (Valerie).

“Mining attracts people that have very dysfunctional relationships with themselves, with their family, with alcohol, sleep, cigarettes … they’re half checked out most of the time ready for the pub to open at night” (Leo).

Blair differed from Leo’s view, noting they had seen mining provide unique opportunities not found in other industries:

“I can tell that most of my colleagues are neurodivergent … they have just fallen through the cracks. And this industry is one of the only industries that pays well for people that have bizarre talents who can learn on the go.”

These views reflect a complex interplay of diverse individuals, industry culture, and external factors, highlighting how prevention strategies that address unwanted sexual attention, coercion and gender harassment must be tailored to this unique context.

Some participants highlighted the diverse range of educational backgrounds and skill sets within the industry, leading to a varied and sometimes challenging workplace culture:

“Usually there are people who come from different educated backgrounds … on site, usually you have less educated people … the main difference is that the technical teams that are on site, they want career development, they’re looking for progress, whereas most of the people in operations are there for the money” (Jordana).

“While we’ve got a lot of diversity in the business in terms of backgrounds and experiences … there’s still an awful lot of uneducated people … there’s lots of people in leadership positions and you can give them all the training in the world, but if they have only ever worked in a workshop, in a big company, in a male dominated industry, then that’s just their normal” (Emma).

The notable presence of older generations who occupied long-standing positions and had accumulated decades of experience within the industry was also highlighted. Some participants observed that these individuals retained deep-seeded beliefs regarding gender stereotypes. Consequently, participants suggested that educational approaches should be customized to effectively address this generation’s perspectives:

“Some of the older people in the industry, they’re having to actually learn how to speak differently, change some of their value systems that have been ingrained in them for a long time … back in the old days where it was all men, you could say whatever you wanted to anyone. I think there’s a feeling of resentment sometimes that can come out in sexual harassment cases because there’s resentment and they’re making jokes to just be like, I can do it, I’m sick of being told what to do” (Lilly).

#### 3.3.5 Infrastructure and environmental challenges

Some physical improvements that mining organizations had implemented to benefit employees were discussed, such as improved lighting and the presence of closed-circuit television cameras (CCTV). However, the physical infrastructure across workplaces and these solutions aimed at supporting employees were thought to be ill-considered at times. For example:

“I feel like most of the things we’re doing are Band-Aid fixes. The business thinks that they’ve solved the issue by putting better doors in with better locks. No, that is such a simplistic view. For me, it starts with language and how women are spoken about then behaviors and the business is doing absolutely nothing to make changes in that space” (Emma).

“If you want to go to the toilet on the floor, you expose your private parts … the cameras are everywhere on mine sites, and the screens where you watch them, anyone can scroll through, zoom in, zoom out. They’ve got those screens in offices, some of them are in private homes, some managers have them in their homes … I was in a meeting where we had this massive screen up and the boss went to the wheel dozer camera … and if the girl who was on the dozer went to go to the toilet you would have seen everything of her private parts, and there would have been 20–30 people watching it” (Valerie).

“When a girl needs to pee, they find an area down a hole … it turns out [my colleague] is on her period often at work and she wants a facility, like a toilet, where she can wash her hands. It wouldn’t even be in question in any other job, in an office or in the city. But she was told she’s being a princess” (Isla).

Furthermore, participants mentioned issues of operational equipment like workplace controls being too high for the average woman’s height and inconveniently placed uniform buttons around the breast area of shirts.

### 3.4 Strategies and programs focused on prevention and response

#### 3.4.1 The effectiveness of in-person training

Participants reported considerable variability in the quality of workplace training focused on SHSA they had received. Some expressed boredom, perceiving training to be a formality rather than a meaningful learning experience. As Joe debated:

“I don’t think many people took much from it … most people just disregarded it and though it was [human resource] bullshit.”

Leo held a similar perspective and highlighted the importance of timing:

“Everyone’s bored shitless … Most people don’t care about safety and most people don’t care about sexual harassment … it happens on a Sunday morning too, so lots of people probably drank too much on a Saturday night.”

Some participants believed that organizational training sessions were a tokenistic gesture to appear proactive about addressing SHSA:

“There was a pamphlet or booklet … it’s not a proper training course, they’re just doing online tick and flick” (Valerie).

Valerie also emphasized how poorly developed and untailored training sessions can yield a counterproductive outcome:

“After the Royal Commission, they did a little bit [of training] but it was presented so poorly. They presented it in a safety meeting, and it was given by a safety rep who was a male, a bit of a tick and flick type person. He presented it with all this bias … the guys were so upset. One guy came up to me and said, I was assaulted by my partner, how come I’m not considered in that? I felt I had to defend why they were doing it because it was presented so poorly, and it made guys look awful.”

Emma expanded on this, stating:

“[Bystander training] is rolled out by superintendents and people who are like, look boys, I know we’ve all heard this 100 times, I’ve just got to get through it. So just sit down and listen, make sure you sign this sheet, and we’ll get through this as quick as we can … on a leader level it’s delivered really well but for the people on the floor, it’s not delivered well.”

However, others appreciated the engagement of actors or interactive methods that encouraged self-reflection and perspective shifts.

The inclusion of real-life scenarios, practical suggestions for bystander intervention, and relatable discussions that emphasized empathy were more likely to resonate with participants and create a positive impact. For instance, some noted that training should be engaging and tailored to various learning styles:

“Even I was bored. And with all due respect to the guys, they’re fitters or processing technicians, they don’t have the concentration span and some of them got lost. But with the actors, even the underground guys have gone you know what that was really good, I got a lot out of that … I find the worst are the guys between 50 and my age … but after they’ve seen some of these actors or had our training, they’ve looked at things in a different light” (Jessie).

“They’d gotten the whole crew together, so like, 80 to 100 people, and they’d done a PowerPoint presentation … I think it actually surprised a lot of people because the number of males [who experienced sexual harassment] was really high as well … they said imagine if it was your wife or your sister or your daughter, how would you feel” (Olivia).

#### 3.4.2 Understanding policy

Even if participants did not know the exact process to follow, most understood that policy documents could be accessed through tools like the search function on the company’s online reporting software or Intranet service. Those who demonstrated better comprehension of these documents were senior leaders or people currently navigating their own reporting of SHSA. However, some participants suggested that people working in operational roles might encounter difficulties in accessing information on reporting:

“I’m not aware of the ins and outs of it to be honest. I’m just aware of the process that I follow through in terms of reporting … I’m not sure I would be able to find it … the crews would find it very difficult to access it” (Joe).

In general, the perception of policy documents was that they appeared static, abstract, and not frequently referred to by employees. For example:

“Most of our policies are quite wordy, they’re obviously written up for legal stuff. They are readily available and there are plenty of people that can explain it if someone doesn’t understand” (Olivia).

These insights suggest a potential disparity between the availability of documents and employees’ comprehension of policy, possibly due to the documents’ perceived complexity or focus on legal compliance.

#### 3.4.3 Management of SHSA

##### 3.4.3.1 Reporting experiences and pathways

Participants openly shared their personal experiences with SHSA, including cases involving friends as victims or perpetrators. Through these shared experiences, a few participants noted improvements in reporting procedures and support for victim/survivors. For example:

“My primary concern with the way it was handled was just that it took such a long time to remove him from the office at the time … Since then, [the company] has done some work to think about what a person-centered response is regarding trauma-informed care” (Ruby).

While companies were perceived to be improving, issues still exist. For example, Lucy discussed the recent outcome of her sexual harassment case in which she experienced unwanted sexual attention. The investigation concluded there was not enough evidence to suggest that the perpetrator sexually harassed her and instead, found that he “acted in an overfamiliar and inappropriate way.” Lucy said:

“Now I have to work with this guy and his team that know he was [eventually] terminated because I spoke up. No one comes out a winner, he lost his job. I’ve gone through a lot of stress and not being able to focus on work and now I’m left to work with his teammates … I’ve had to live with the repercussions of speaking out, and that’s the hard thing. It takes a lot of courage and bravery to speak out when something inappropriate happens.”

Most participants were familiar with reporting procedures implemented in their workplaces. However, there were perceived shortcomings in the over-reliance on line-managers as the main point of contact with a few participants noting this could result in victims feeling uncomfortable to report to their leaders about work colleagues. For instance, Freya expressed:

“As a female, I don’t know how many times in a meeting I get spoken over by a male. So why would I report to them? Why would I want to go and talk to them about something that has actually made me feel very vulnerable when you talk over me at a meeting?” (Freya).

In another example, Emma shared an incident when she was asked to participate in an investigative process because the management team was uncomfortable discussing “girl stuff.” After the victim’s interview, Emma recalled a conversation between a superintendent and a manager who both speculated that the victim’s aim was to avoid her ex-partner, implying the incident was not significant. However, it was eventually deemed serious enough for police involvement. Emma expressed concerns that incidents might not be taken seriously if investigated by the “boys club.” Other women also used this term to describe the challenges employees might face when reporting, especially if the alleged perpetrator had long tenure with the company, suggesting the prevalence of gendered harassment within the industry. Men also expressed disappointment in reporting processes:

“I think [being afraid to report] is the main issue … there’s an extra level of difficulty when working in mining because there’s no escape. Once you’ve reported, it’s usually a colleague you have to work with … the victim has to think about, if I report this guy, I’ve got to work with him. I imagine that’s a very difficult decision to make and a real obstacle to reporting” (Bob).

Furthermore, participants voiced concerns about companies’ reliance on the absence of reports as an indicator of successful prevention measures:

“The impetus is often on the victims, so if people aren’t reporting things or feeling comfortable enough to report things, nothing can happen. Companies will often say, we haven’t had any reports of things, so we must be doing really well. And I’ve made a few suggestions for that not to be the primary metric of how many reports you get” (Clara).

There was a disparity with participants’ knowledge of the ways in which company policies or reporting tools can be accessed. Some participants believed that policy and reporting tools were available solely on work computers and could not be accessed on personal devices. For example, Emma detailed:

“To get to the digital workspace you have to be on a work computer. I feel like that could actually be a barrier because for a lot of people on the floor you might not actually have access to a work computer to get into it, and if you do have access, a lot of them are in the crib areas or open spaces where anyone could walk past and look over your shoulder and be like, oh what are you doing on [the company’s online reporting service]. So yes, we can access it relevantly easily on the face of [the desktop] but the practical application is I think, quite limited and hasn’t really been considered.”

Other participants had conflicting knowledge about this. For instance, Liam spoke on being “pretty confident” that he was able to access policy and the company’s online reporting service from a home device. However, others expressed uncertainty regarding accessibility on personal devices. Consequently, knowledge varied:

“You can’t do it on your home computer because you won’t have the log in and VPN, but we can have a work profile on our phone. I think it’s available from phones … doing a long reporting thing on an iPad or phone is not ideal. I had never thought of that, I feel a bit sad that some people don’t have access to [the company’s online reporting service] in their own time off. That’s weird that they have to do it on work time, that’s ridiculous” (Blair).

Another participant, Amelia, was a leader and had seen the “back end” of the reporting process, highlighting issues with anonymous reporting methods:

“We’ve also got a speak out line that people can contact anonymously, but we know it’s not anonymous … because it kind of can’t be. If someone has got a problem that you need to solve for them, not for the broader population, it’s at the leader’s discretion whether they would go straight to human resources, who would then engage with the one-up leader, and they would start investigating it, collecting information and speaking to that person. It comes down to the integrity of that leader, whether they disclose the identity of that person or not”.

##### 3.4.3.2 Unfair dismissal of sexual harassment

A few participants shared experiences of colleagues who they perceived had been unfairly dismissed and removed from site, from what they considered to be less serious or non-physical offences of gender harassment and sexual harassment. They believed these colleagues had faced unwarranted dismissals due to the heightened awareness and stricter regulations surrounding SHSA. All participants understood that sexual assault (and rape) were criminal behaviors that warranted police investigations and the immediate removal of perpetrators, but some participants articulated responses to gender and sexual harassment incidents had been overly punitive.

Some participants were concerned that excessive penalties were being imposed, leading to hyper-vigilance and general uneasiness. Freya illustrated this:

“A guy pulled a girl’s ponytail and someone else saw that in jest and felt that was inappropriate and reported it. And that guy got absolutely grilled, even though the woman who was involved with the pulling of the ponytail was like ‘oh, we were just mucking around with each other, we have been for 10 years type of thing’. But the company went too far [in reprimanding him] and that guy was like, I’m out of here. I’m not going to get treated like that”.

Participants discussed the potential benefits of providing rehabilitation and education for alleged perpetrators to build their knowledge on gender harassment, rather than solely resorting to immediate removal:

“I do wish there was more investigation or seeing his side and understanding the context a bit more, rather than just concluding that it is harassment without properly understanding the context” (James).

Jordana also provided an insightful perspective on what she deemed a “perfect situation” for handling such incidents:

“Put him or her under mentoring and train them so it’s not something like, now we’re going to sit down and have an investigation. We have to move away from this, especially in the low-level events … just give a chance to the perpetrator to be better in the next situation”.

Here, Jordana highlighted the potential for rehabilitation and learning to prevent future incidents, particularly for cases perceived as lower in severity.

Participants noted that expecting all witnesses and bystanders to report incidents could be problematic when there is the potential for colleagues to lose their jobs. James highlighted that the current approach of “see something, say something” did not consider all elements of the issue:

“While on face value that sounds like a no brainer, they’ve got to think of the implications. How’s it going to affect the reporter? How’s it going to affect the people who you’re talking about? Could it be handled at a much lower level? As soon as human resources get involved, they’re there to make a name for themselves.”

However, some of these participants suggested that dismissed employees might relocate to another organization without facing repercussions. Some believed the current system lacked meaningful consequences, allowing perpetrators to continue their harmful behavior elsewhere, potentially leading to increased resentment. Emma spoke about the need for broader cultural change and improved communication:

“A cultural shift needs to happen … it’s either been like, these people are moved elsewhere so the noise goes away or it’s that the investigation has been completed and not substantiated … there’s so much resentment towards women taking their jobs, so there’s no respect. Then, poor behaviors ensue. I think there could be better communication, not just education”.

##### 3.4.3.3 Reporting of incidents to the broader workplace

Participants’ accounts revealed a noticeable effort within the mining industry, especially among larger companies, to implement workplace changes. A common approach involved addressing physical barriers first, then implementing SHSA policies, and finally integrating training initiatives into the workforce. However, participants highlighted a notable difference in the dissemination of information regarding psychosocial incidents, especially those related to SHSA, compared to safety incidents. While safety incidents prompted swift and widespread alerts throughout the company, responses to SHSA incidents varied considerably. Some participants noted that organizations chose to keep such incidents secret, with one participant stating:

“There was a lot of secrecy about it… I think that keeping things secret makes it easier for the perpetrators to do the same thing but as a different site” (Clara).

The issue of secrecy surrounding incidents of unwanted sexual attention, coercion, and gender harassment was highlighted by participants as a potential catalyst for workplace gossip and inappropriate discussions. One participant recounted an incident that occurred inside an airport illustrating this:

“One of the leaders who is a woman felt the need to tell us that somebody had gotten raped at camp, which clearly, since nobody else had shared that with us, it was not something she was supposed to tell us, especially since we’re on the frontline and she was a leader. She then proceeded to make incredibly distressing … victim blaming statements saying, oh but do you see some of the women what they wear at the wet mess? Basically, implying that the way you dress makes you more susceptible to rape” (Blair).

A few participants, mostly those in leadership positions, shared experiences of receiving “safety banners” or email alerts from organizational leaders when psychosocial hazards like SHSA occurred. These communications aim to provide “confidential” and “informative” details about the incident and its classification as SHSA. Such alerts were often discussed in meetings to foster dialogue and understanding. However, the sensitive nature of SHSA reporting sometimes led to slower communication, as these alerts were “manicured” and underwent inspection by various departments. For example:

“It’s perfect and has been written by a group of people, including human resources to make sure it doesn’t reveal identities. So, the reporting is slower than other types of reporting because it’s going to be scrutinized by the workforce” (Amelia).

Such emails or safety banners aim to counteract workplace gossip by “stopping all the rumors” (Eleanor) and spotlight real-life experiences within the organization. Nevertheless, using this method to disseminate information about SHSA raised concern about the potential for vicarious trauma. While the use of safety banners was described as “relatively new,” participants had already seen the impacts of trauma:

“Yes, I’ve seen females, only females, crying, and I’ve cried at them as well. [SHSA] is still going on. This is real. People are living in hell, you know, with these situations” (Amelia).

As Amelia identified, the sudden reception of emails during the workday could trigger emotional reactions and as a result, employees could experience distress.

## 4 Discussion

Using Fitzgerald’s Tripartite Model of Sexual Harassment, the interconnected nature of SHSA in the mining industry was explored (7, 8). This research carries significance in engaging mining employees as key stakeholders in preventing SHSA within the industry, fostering a collaborative platform between communities, employees, and mining organizations (30). The risks and challenges related to SHSA have been discussed, offering implications for future prevention strategies tailored to the industry’s unique context. The findings indicated improvements in workplace culture over recent years. However, in line with the Tripartite Model, many participants observed that subtler forms of gender harassment are more common than unwanted sexual attention and coercion, as well as sexual assault (7, 8). Research has highlighted variations in the types of SHSA behaviors that occur (16, 31), with these behaviors evolving into more subtle and nuanced forms over time (32). For instance, in a Canadian study of women’s experiences in the oil and gas industry, participants spoke on changing their behaviors and adopting less feminine traits to meet industry demand for “toughness” (10). This pattern of workers experiencing “everyday” forms of sexism has been highlighted in other industries, such as construction (33).

Some participants expressed perspectives reflecting rape myths, which are misconceptions and stereotypes about sexual violence that can normalize and justify SHSA (34). Indeed, this finding supports previous research that has identified the prevalence of hostile sexism within masculine-dominated work environments, such as the military (35). In the current research, these beliefs were particularly relevant to notions regarding personal appearance and its impact on employees’ treatment and interactions. For instance, some participants suggested that women should avoid wearing leisure or gym attire in dining halls, reflecting unwanted sexual attention (7, 8), shifting responsibility onto potential victims rather than addressing misconduct or harassment directly (36). Although research on the prevalence of rape myths within the mining industry is scarce, personal statements from victim/survivors in the *Enough is Enough* report (16) revealed adjustments in clothing choices on-site due to the large number of male employees. These findings are significant, as they highlight the need for the adaptation and refinement of workplace training that is specific to the nuanced experiences of mining industry workers. It also highlights the need for future research to explore how covert, deceptive, overt, and explicit behaviors are interrelated and manifest within the workplace. Future research should further use Fitzgerald’s Tripartite Model (7, 8) to understand how unwanted sexual attention, coercion, and gender harassment are prevalent within the industry. Gathering these insights will be critical to the development of relevant and effective workplace training that aims to prevent SHSA.

The study revealed that mining organizations have implemented various initiatives to promote gender equality and reduce gender harassment in the workplace, including the common practice of gender ratios, particularly in larger mining organizations. However, some participants mentioned that women were sometimes perceived as “diversity hires,” suggesting that organizations prioritize gender representation over skill suitability. This observation aligns with findings by Campero et al. (37), which highlighted how including women in the mining industry was often correlated with increased profitability for companies. However, hiring women without ensuring they possess the necessary qualifications or providing adequate skill development training may perpetuate stigma and reinforce the notion that women are unsuitable for roles in the industry. This could ultimately undermine the accomplishments of women who have earned their positions based on merit. Thus, there appears to be a gap between the industry meeting equality targets and creating an equitable workforce (37). This balance represents an ongoing challenge; however, the findings of this research highlight the importance of gender equality for safety, especially on remote sites. The need for more women in leadership roles to advocate for positive changes to workplace culture was also seen as essential (38, 39).

Participants detailed that despite some progress, fundamental requirements for women’s comfort may still not be adequately addressed in the mining industry. Specifically, concerns were raised regarding the lack of bathroom facilities for women and concerns over people in office spaces who monitor CCTV cameras being able to observe employees urinating. These issues underscore both gender disparities in workplace infrastructure and the need for organizations to prioritize the needs of all employees, fostering an equitable and safe workplace. Botha (1) suggested that structural barriers in the mining industry could primarily stem from its historically male-oriented nature. Their study highlighted challenges faced by women, particularly concerning the inadequate provision of suitable ablution facilities especially for those working underground (1). Poor infrastructure, such as a lack of inclusive bathrooms and invasive CCTV usage, contributes to an environment that may not be considered for all employees. While infrastructural changes like underground bathroom access may be cumbersome to achieve, findings from this study assert that physical and structural changes are essential first steps to ensuring that employees feel safe and accounted for.

Standardizing and enhancing training for all employees regardless of their position within the organization was perceived to be a crucial step in increasing awareness and preventing SHSA. Participants stressed the need for training programs that are authentic rather than tokenistic, challenged gender harassment and recognized men as potential victims of SHSA. Our findings extend recent research which asserts that Western Australian mining employees typically receive limited training on SHSA, often delivered as part of the orientation process while introducing the company’s policies (22). Although Kalinosky (40) and Zelin and Magley (41), and others note that training is not always effectively implemented, more recent studies highlight the importance of adopting evidence-based approaches to enhance workplace training (42–45). Participants in this study called for training opportunities that are engaging and relevant, focus on and challenge gendered stereotypes and norms, and recognize men as potential victim/survivors of SHSA. Furthermore, the inclusion of trained educators who facilitate role-playing exercises and hearing from lived experiences were also considered effective training practices. Importantly, including mining employees from diverse roles in the development and implementation of workplace training was perceived to improve trustworthiness, relevancy, and impact. In effect, workplace training must address the persistence of gender harassment presented in Fitzgerald’s Tripartite Model to prevent SHSA (7, 8).

Participants in this study described policy documents as rarely accessed and static. Similarly, a recent study in the Western Australian mining sector (22) found that policies were developed without input from workers who were most likely to need and refer to them. The findings of this research highlight the importance of co-design where mining industry workers from different roles are involved in the development and implementation of site customized policy documents, to ensure they are accessible, understandable and relevant to the context of each workforce. The promotion of and education about these documents is also required.

Some participants discussed the challenge of measuring the effectiveness of prevention measures by changes in incident reports. Positive shifts in workplace culture are likely to increase awareness and confidence in reporting. Further to this, employees may opt to report incidents of SHSA externally rather than internally, impacting incident rates. Additionally, participants detailed that reporting could become difficult when anonymity cannot be guaranteed. This issue may be more prevalent in the mining industry due to close-working teams and remote work environments. Indeed, the experiences of victims/survivors reporting incidents of SHSA vary widely. The findings suggest that improvements to reporting systems must be made with attention to the nuanced landscape of the mining industry. Specifically, there is potential to enhance anonymous reporting options to protect employees’ identities and mitigate tensions between victim/survivors and their work teams. While anonymous reporting systems can safeguard individuals’ identities, they may also limit the ability to thoroughly investigate incidents and take appropriate actions, as demonstrated in some workplace bullying literature (46). Therefore, there is a need for future research to explore the provision and perceived impact of anonymous reporting options within the mining industry.

While some participants reported their experiences were minimized or excused by senior leaders, others spoke of employees being hesitant as they feared their report might lead to immediate dismissal of the perpetrator/colleague. While a “zero-tolerance” approach may be suitable for some incidents, sole use of this approach limits opportunities for educational prevention strategies and restorative actions (47) and may also lead to underreporting. It is evident that organizations must shift their main focus from a “punishment” to “repair” perspective, considering individual incidents and working to understand the needs of all parties involved. Responding to incidents in more proportionate and appropriate ways that include educational prevention strategies (31) rather than relying solely on punitive measures will be crucial to shifting employee attitudes and behaviors. This will ultimately contribute to more positive workplace cultures.

## 5 Strengths, limitations, and future research opportunities

This study is significant as there are limited qualitative investigations into SHSA within the Western Australian mining context. It included a range of mining organization sizes (small, medium, and large), participants from various roles within the industry, and a diverse range of people including men, women, and a nonbinary individual. Random assignment of participants to interview groups within the larger project (covering SHSA, mental health, and emerging safety issues) (25) also enhanced the likelihood of capturing a broader spectrum of perspectives. This approach encouraged the participation of workers who might not otherwise engage in discussions of SHSA or may feel less knowledgeable about the issue and therefore, not have expressed their interest to participate.

The research included participants across various age groups, although more representation of young adults (aged 18–35) entering the mining industry could have offered valuable insights, particularly considering their heightened risk of experiencing sexual harassment (14). While the study engaged workers who had experienced positive workplace transitions, exploring the perspectives of those entering post-transition would have enriched the findings. Understanding the perspectives of young men is also crucial for shaping prevention strategies, given their significant presence in the mining workforce (48). Furthermore, SHSA behaviors can sometimes intersect with other issues like bullying, potentially leading to participants struggling to distinguish between these issues (14, 16). Despite this challenge, the interviews included participants defining SHSA, with interviewers elaborating on their definitions to guide the discussions effectively.

While this initial qualitative investigation has shown a willingness among the workforce to discuss these topics and has drawn from diverse perspectives, more purposeful data collection efforts may reveal specific insights and perspectives. Moving forward, it is essential to collect further qualitative data from specific groups to gain a deeper understanding of SHSA within the industry. This includes gathering perspectives from victim/survivors, perpetrators, alleged perpetrators, Work Health and Safety staff who receive reports, First Nations groups, people living with disability, people from culturally and linguistically diverse backgrounds, and members of the LGBTQIA+ community. Additionally, while the participants had received some level of training on SHSA, this varied considerably in its effectiveness and approach. Thus, future research could further explore the efficacy and effectiveness of workplace training mining employees receive. As the industry evolves in its approaches to addressing SHSA, it will be crucial to continue gathering perspectives from mining employees and repeating the data collection process over time to document any changes that occur. This ongoing research will contribute to a more comprehensive understanding of SHSA dynamics within the mining industry and inform the development of effective prevention strategies.

## 6 Conclusion

Scholarly understandings of SHSA in the Western Australian mining industry is currently limited, despite recent inquiries highlighting its prevalence. Engaging mining employees as key stakeholders is crucial to advancing understandings of this issue and prevention efforts. This research used Fitzgerald’s Tripartite Model of Sexual Harassment to offer insights into mining workers’ understandings of SHSA and workplace training and policies. While participants noted improvements, this study revealed ongoing risks and challenges associated with SHSA in the mining industry. These findings highlight the need for continued development and implementation of prevention strategies that improve responses to and recognition of SHSA in the industry.

## Data availability statement

The datasets presented in this article are not readily available to maintain the privacy and confidentiality of the participants. Requests to access the datasets should be directed to jacqui.hendriks@curtin.edu.au.

## Ethics statement

The studies involving humans were approved by the Curtin University Human Research Ethics Committee (HREC2022-0502). The studies were conducted in accordance with the local legislation and institutional requirements. The participants provided their written informed consent to participate in this study.

## Author contributions

SV: Conceptualization, Formal analysis, Investigation, Writing – original draft, Writing – review & editing. SB: Conceptualization, Formal analysis, Methodology, Project administration, Supervision, Writing – original draft, Writing – review & editing. CY: Conceptualization, Funding acquisition, Methodology, Project administration, Writing – review & editing. SP: Conceptualization, Funding acquisition, Methodology, Writing – review & editing. JH: Conceptualization, Formal analysis, Methodology, Project administration, Supervision, Writing – original draft, Writing – review & editing.
