# Supplementary material for: Sexual harassment and sexual assault in the Western Australian mining industry: a qualitative examination of the perceptions of key stakeholders
Source: Front Public Health. 2024 Aug 8;12:1432990. doi: 10.3389/fpubh.2024.1432990 (PMC11342336; doi:10.3389/fpubh.2024.1432990)
Supplement: Supplementary file 1 [file Table_1.docx]

***Supplementary Material***

**Sexual harassment and sexual assault in the Western Australian mining industry: A qualitative examination of the perceptions of key stakeholders**

**Sarah Vrankovich^1^, Sharyn Burns^1^, Cheryl M H Yam^2^, Sharon K Parker,^2^ Jacqueline Hendriks*^1^**

*** Correspondence:**

Jacqueline Hendriks

* [jacqui.hendriks@curtin.edu.au](mailto:jacqui.hendriks@curtin.edu.au)

**Demographics**

We are looking to interview people with a mix of genders, ages, job roles, companies, and years of work in mining. Could I please collect this information from you, just for the purpose of trying to ensure we interview a variety of people?

Are you comfortable if I turn on the recording? Thank you.

**Workplace Background**

1. Could you please tell me a little about the work you do. What is your role?

1. How would you describe your workplace culture?
2. Reflecting on your gender, what is your experience of working in the mining industry? Do you think this would be different if you were a different gender? How so?

**Sexual harassment and sexual assault** 
We are going to be speaking about sexual harassment and sexual assault specifically now.

1. Thinking about the terms, sexual harassment and sexual assault, could you please tell me what you think they mean?

Great! To build on this definition, we would like to add:

***Sexual harassment and assault.*** *These are terms used to describe any sexual activity that happens without consent. Harassment may include the following:*

- *unwanted sexual advances,*
- *offensive jokes,*
- *sharing unwanted sexual material,*
- *sexual innuendo,*
- *staring,*
- *requests for sex or implied requests*
- ***And sexual assault include*** *unwelcome sexual contact such as groping, touching, and ‘next-level’ behaviour such as stalking and rape.*

*It’s important to remember that harassment can also occur in online spaces. Sexual harassment and sexual assault can also attract criminal prosecution.*

1. What is your impression of sexual harassment and sexual assault in your workplace? (How common do you think sexual harassment and sexual assault it is in your workplace?)

1. Do you think that there are unique challenges of working within the mining industry that impact how common sexual harassment and sexual assault are?

1. How does your workplace respond to incidences of sexual harassment and sexual assault in the workplace? (Have you experienced either of these firsthand or witnessed them?)

(If relevant) Have responses been adequate?

1. Have responses to sexual harassment and sexual assault altered during your working life within the mining sector, and how so? Have responses gotten better or worse?

**Strategies and initiatives**

1. Are you aware of your company’s policies around sexual harassment? How accessible are these policies? How well are they implemented?

1. Has your workplace implemented or provided any other strategies or programs to reduce sexual harassment and sexual assault, and improve workplace culture? How effective do you find these strategies? How well are they received by staff?

(If required) Some examples might include workplace training, reporting guidelines, factsheets, support services.

1. In terms of reducing incidents of sexual harassment and sexual assault, are there any strategies that you can suggest for mining workers specifically? What about for improving workplace culture? What would work best as an appropriate intervention in your mind?

1. Is there anything that we haven’t covered that you would like to add?

Thank you for your time.
